# New Metastable Baro- and Deformation-Induced Phases in Ferromagnetic Shape Memory Ni_2_MnGa-Based Alloys

**DOI:** 10.3390/ma15062277

**Published:** 2022-03-19

**Authors:** Vladimir Pushin, Alexander Korolyov, Nataliya Kuranova, Elena Marchenkova, Yurii Ustyugov

**Affiliations:** Laboratory of Non-Ferrous Alloys, M.N. Miheev Institute of Metal Physics of Ural Branch of Russian Academy of Sciences, 620108 Ekaterinburg, Russia; korolyov@imp.uran.ru (A.K.); kuranova@imp.uran.ru (N.K.); elenamarch@imp.uran.ru (E.M.); ustyugov@imp.uran.ru (Y.U.)

**Keywords:** Heusler alloy Ni_50_Mn_28.5_Ga_21.5_, martensitic transformations, megaplastic (severe) deformation, atomic disordering, nanostructure, properties

## Abstract

Structural and phase transformations in the microstructure and new metastable baro- and deformation-induced phases of the Ni_50_Mn_28.5_Ga_21.5_ alloy, typical of the unique class of ferromagnetic shape memory Heusler alloys, have been systematically studied for the first time. Phase X-ray diffraction analysis, transmission and scanning electron microscopy, and temperature measurements of electrical resistivity and magnetic characteristics in strong magnetic fields were used. It was found that in the course of increasing the pressure from 3 to 12 GPa, the metastable long-period structure of martensite modulated according to the 10*M*-type experienced transformation into a final non-modulated 2*M* structure. It is proved that severe shear deformation by high pressure torsion (HPT) entails grainsize refinement to a nanocrystalline and partially amorphized state in the polycrystalline structure of the martensitic alloy. In this case, an HPT shear of five revolutions under pressure of 3 GPa provided total atomic disordering and a stepwise structural-phase transformation (SPT) according to the scheme 10*M* → 2*M* → *B*2 + *A*2, whereas under pressure of 5 GPa the SPT took place according to the scheme 10*M* → 2*M* → *B*2 → *A*1. It is shown that low-temperature annealing at a temperature of 573 K caused the amorphous phase to undergo devitrification, and annealing at 623–773 K entailed recrystallization with the restoration of the *L*2_1_ superstructure in the final ultrafine-grained state. The size effect of suppression of the martensitic transformation in an austenitic alloy with a critical grain size of less than 100 nm at cooling to 120 K was determined. It was established that after annealing at 773 K, a narrow-hysteresis thermoelastic martensitic transformation was restored in a plastic ultrafine-grained alloy with the formation of 10*M* and 14*M* martensite at temperatures close to those characteristic of the cast prototype of the alloy.

## 1. Introduction

Alloys undergoing thermoelastic martensitic phase transformations (TMPTs) have a great innovative potential for a variety of structural and functional applications due to the effects of shape memory (SM), giant superelasticity (GS), elastic- and magnetocaloric (EMC) and other phenomena [1,2,3,4,5,6,7,8,9,10]. Notable among these alloys, of course, are the atomically ordered *L*2_1_ Heusler alloys, which exhibit a ferromagnetic ordering below the Curie temperature *T_C_* [10,11,12,13,14,15,16,17,18,19]. For alloy compositions with the electron concentration *e*/*a* < 7.7, TMPT occurs from a ferromagnetic parent phase (i.e., *M_s_* < *T_C_*, where *M_s_* is the temperature at the start of martensitic transformation). A unique specific feature of Heusler ferromagnetic alloys based on the Ni-Mn-Ga system is the ability to control the TMPT, SM, GS, and EMC effects not only by temperature and external mechanical forces, as in other alloys [1,2,3,4,5,6,7,8,9], but also by the magnetic field [5,10,11,12,13,14,15]. During cooling below *M_s_*, these alloys exhibit a sequence of first-order phase transitions from the parent cubic *L*2_1_ phase to long-period modulated intermediate martensite structures (denoted as 10*M* and 14*M*), as well as to tetragonal 2*M* martensite without lattice modulation [5]. A significant key limitation for wide practical application is the brittleness and poor machinability of ferromagnetic and other polycrystalline SM alloys, with the exception of titanium nickelide [1,2,3,4,16,18,20]. The high brittleness of Ni_2_MnGa-based alloys even in a single-crystalline state represents an obvious obstacle for realization of SM, GS, and other related effects.

There exist several ways to improve the ductility of intermetallics, among which grain size reduction and atomic disordering should be mentioned [5,18]. It has been established that the combined technologies of ultra-rapid quenching (URQ) from the melt [21,22,23,24,25,26,27,28,29,30] and severe plastic deformation (SPD) [31,32,33,34,35,36] can provide production of SM alloys of individual chemical compositions based on titanium nickelide and copper, in a high-strength and ductile fine-grained (FG) [or ultra-fine-grained (UFG)] state. A URQ from the melt entails an increase in the strength and ductility of FG alloys with a mean grain size in the interval 0.2–1.0 μm, depending on the regime of subsequent thermal treatment. SPD provides for the formation of nanocrystalline UFG structure of the alloys (with the size of nanograins less than 100 nm). This leads to their strong hardening, but entails a decrease in their ductility. However, the ductility of these SPD UFG alloys can be significantly improved by subsequent low-temperature short-term annealing. An understanding of the combined treatment under discussion has been successful in (for example) the creation of wire demonstrating high strength and good plasticity, and of rods or sheets with SME for alloys based on titanium nickelide and copper. Recently, work on the creation of UFG structures using these methods of extreme external influences was performed on Ni_2_MnGa Heusler alloys by URQ from the melt [37,38,39,40,41,42,43], magnetron sputtering techniques [44,45] or the SPD methods [24,46,47,48,49], including ball milling [50].

In the present article, a comprehensive systematic analysis of structural-phase transformations was carried out for the first time in the three-component SM Ni_2_MnGa-based alloy, subjected to combined SPD by high-pressure compression (HPC) and subsequent high-pressure torsion (HPT), as well as heat treatment. This work studies the influence of applied HPC and HPT on the transformation behavior and evolution of physical electrical and magnetic properties in the investigated low elastic-modulus Ni_50_Mn_28.5_Ga_21.5_ alloy.

## 2. Materials and Methods

The Ni_50_Mn_28.5_Ga_21.5_ alloy was melted out from nickel, manganese, and gallium of 99.99% purity in an electric arc furnace (Institute of Metal Physics, Ekaterinburg, Russia) in a helium atmosphere with triple remelting and subsequent long-term homogenizing annealing in the temperature range of 1073–1173 K. The chemical composition of the alloy according to integral spectral analysis was Ni–28.50 at.% Mn–21.50 at.% Ga. The grain sizes of the cast alloy reached several millimeters.

Samples for deformation in Bridgman anvils were made in the form of disks with a diameter (*D*) of up to 10 mm and a thickness (*t*) of 0.5 mm. The amplitude of high pressure was determined by the ratio of the value of applied load and the square of the anvil. True logarithmic deformation was determined as *e* = ln(*t*_i_/*t*_f_) + ln(π*nD*/*t*_f_), where *t*_i_ and *t*_f_ are the initial and the final thickness (of the sample), *n* is the number of revolutions. HPC deformation was performed at 3 and 12 GPa, and HPT deformation was performed at 3 GPa and five revolutions, and at 5 GPa and two and five revolutions HPC and HPT were carried out at room temperature (RT). The subsequent isothermal vacuum-sustained annealing was carried out for 10 min at temperatures in the interval of 373–973 K.

The phase composition and structural-phase transformations were studied by X-ray diffraction (XRD, Empyrean “PANalytical”, in monochromatized CuKα radiation) (Malvern Panalytical B.V, Almelo, The Netherlands), analytical scanning (SEM, Quanta 200 Pegasus at 30 kV) (FEI Europe B.v., Eindhoven, The Netherlands) and transmission electron (high-resolution TEM, Tecnai G^2^ 30 and CM30 at 300 kV) (FEI Europe B.v., Eindhoven, The Netherlands) microscopy, including in situ investigations under heating or cooling.

The temperature measurements of electrical resistivity and magnetic characteristics were carried out by the potentiometric method, on the MPMS-5XL SQUID magnetometer (Quantum Design, San Diego, CA, USA) and on the PPMS-9 installation (Quantum Design, San Diego, CA, USA), and the temperatures at the start (*M_s_*, *A_s_*) and finish (*M_f_*, *A_f_*) of the forward (*M_s_*, *M_f_*) and reverse (*A_s_*, *A_f_*) TMPTs of the alloy were determined using the two tangent method.

## 3. Results and Discussion

**Figure 1:**
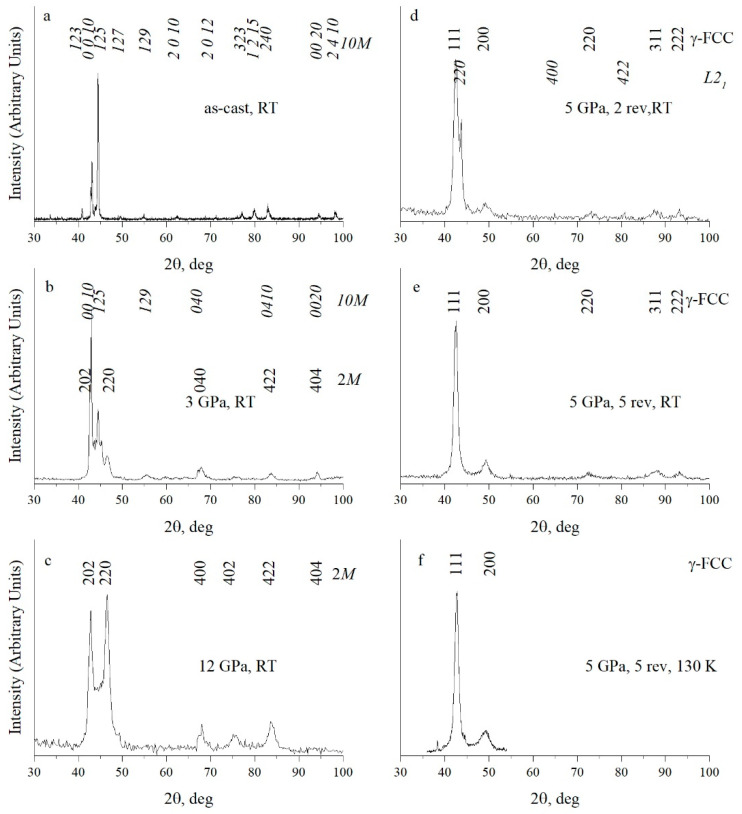
X-ray diffractograms of Ni_50_Mn_28.5_Ga_21.5_ alloy (**a**) in the initial cast state; (**b**) after HPC at 3 GPa, (**c**) at 12 GPa; after HPT at 5 GPa at (**d**) 2 and at (**e**,**f**) five revolutions. The observations were performed at (**a**–**e**) RT and (**f**) 130 K.

According to the XRD analysis data, the initial cast coarse-grained alloy Ni_50_Mn_285_Ga_21.5_ after synthesis was in a state of modulated long-period 10*M* martensite (Figure 1a, Table 1) [51,52].

**Figure 2:**
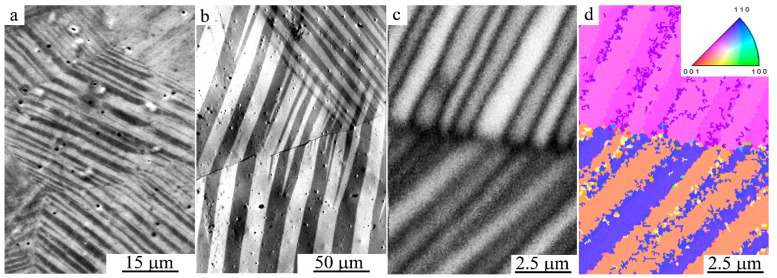
SEM images of (**a**) the packet microstructure of 10*M* martensite in the initial cast alloy Ni_50_Mn_28.5_Ga_21.5_ in secondary electrons; (**b**) magnetic domains under Lorentz microscopy, and (**c**,**d**) microcrystals with a thickness of ~1 micron of twin orientation according to EBSD.

Figure 2 shows the hierarchy of the packet micromorphology of 10*M* martensite in the cast alloy. It is seen that the packet micromorphology is formed by primary microcrystals (Figure 2a), twinned pairwise according to the data from electron back-scattered diffraction (EBSD) (Figure 2c,d), and contains larger magnetic microdomains in accordance with Lorentz microscopy (Figure 2b).

**Figure 3:**
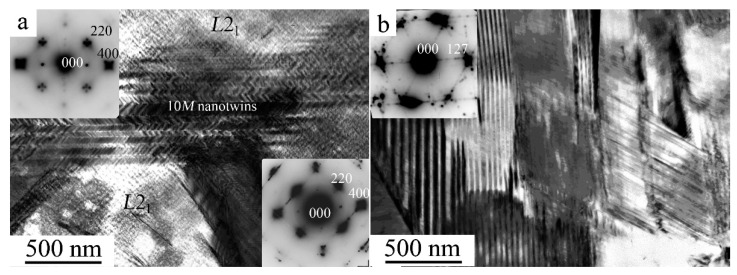
Bright-field TEM images of the microstructure of the cast alloy Ni_50_Mn_28.5_Ga_21.5_ at (**a**) RT and (**b**) 120 K and the corresponding micro electron diffraction patterns (SAED, in the inserts). The SAED patterns correspond to residual *L*2_1_ austenite (zone axes (a.z.) [010] *L*2_1_, insert on the left in (**a**), thin nanotwins of the 10*M* (a.z. [010] 10*M*, insert on the right in (**a**) and the 14*M* (a.z. [111] former *L*2_1_, insert in (**b**) martensite. The observations were performed at (**a**) RT and (**b**) 120 K.

Figure 3 shows that, according to TEM data, secondary nanotwins were present inside individual martensite crystals. Figure 3a demonstrates the mechanism of intragrain formation of a packet of nano-twinned 10*M* martensite crystals in a TEM in situ experiment after heating by an electron beam. In the regions of the residual initial high-temperature *L*2_1_ phase, a typical pre-martensitic tweed contrast is visible, accompanied by the appearance of diffuse streaks along <110> and satellites in positions of type 1/6 <220> *L*2_1_ in the SAED patterns (see Figure 3a). Cooling in situ TEM from RT to 120 K led to the second TMPT with the formation of nano-twinned long-period 14*M* martensite packets (Figure 3b) [52,53,54].

According to the XRD data, HPC at 3 and 12 GPa affected the phase composition of the alloy (Figure 1b,c), leading to partial (after 3 GPa) or complete TMPT (after 12 GPa) into the so-called non-modulated martensite with tetragonal structure of type 2*M* (Table 1). The grain sizes, as a rule, did not change, despite the tenfold excess of pressure at HPC over the strength limit of this low-modulus alloy. An application of HPT under a pressure of 5 GPa and two and five revolutions caused further radical phase changes with formation in the alloy—according to the XRD analysis—of the γ-FCC structure (of type *A*1) (see Figure 1d–f; and Table 1). At the same time, after HPT for two revolutions, the preservation of traces of the *L*2_1_ phase was noted (Figure 1d, Table 1).

**Figure 4:**
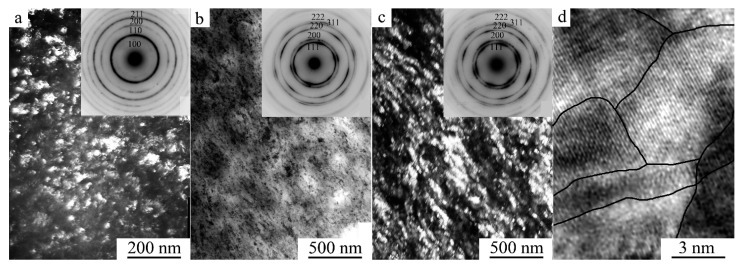
(**a**,**c**) Dark- and (**b**) bright-field TEM images of the nanostructure, and (**d**) a direct resolution image of the atomic structure of the Ni_50_Mn_28.5_Ga_21.5_ alloy subjected to HPT at five revolutions at (**a**) 3 GPa and (**b**–**d**) 5 GPa, and corresponding SAED patterns (in the inserts). The observations were performed (**a**,**b**,**d**) at RT and (**c**), insert in (**a**) at 120 K.

According to the TEM data, HPT under a pressure of 3 GPa and five revolutions completely changed the microstructure of the alloy (Figure 4a). The dark-field TEM image of the alloy structure shows that the sizes of nano crystallites were 10–20 nm. When cooled to 120 K in the mode of TEM in situ, the dimensional and morphological features of the nanostructured *B*2 state in the alloy were preserved (insert in Figure 4a). The reflections in the SAED pattern (see the insert in Figure 4a) were distributed over the rings and had the following *hkl B*2-phase indices: 100, 110, 200, 211, etc. Moreover, judging by the halo—a weaker individual diffusive ring near the 100 position—the alloy was in a partially amorphous state at cooling up to 120 K. Methodically reliable identification of the β phases *L*2_1_, *B*2, and *A*2 was provided only by visualization of weak superstructural reflections in the SAED patterns of the types 111 and 200 from *L*2_1_, 100 from *B*2, or their absence for the *A*2 structure. In this case, we can only conclude unequivocally that the (*B*2 + *A*2) nanostructure is dominant in the alloy, since super-structure reflection of 111 *L*2_1_ type was absent in the SAED patterns.

After HPT at a pressure of 5 GPa and five revolutions, similar TEM images of the nanostructured state of the alloy were observed. However, SAED patterns had fundamentally changed (inserts in Figure 4b,c). According to the results of their indexing, in accordance with the XRD analysis (Figure 1e,f) the alloy has a structure of γ-FCC up to 120 K (of type *A*1: with *hkl* indices 111, 200, 220, 311, etc.). It can be assumed that the amorphous component detected due to continuous diffuse halos (inserts in Figure 4a–c) was localized on the blurred, sinuous intercrystalline interfaces of γ nanocrystals clearly visualized in direct atomic resolution images (Figure 4d). It is along these that the bending contours of extinction were localized in TEM images.

**Figure 5:**
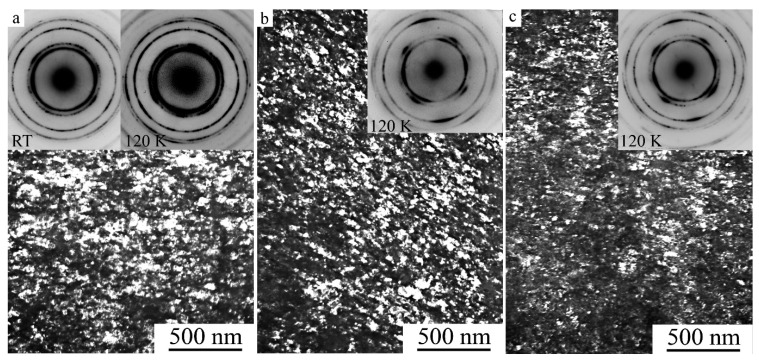
(**a**–**c**) Dark-field TEM images of the microstructure and corresponding SAED patterns (in the inserts) of the Ni_50_Mn_28.5_Ga_21.5_ alloy subjected to HPT at 5 GPa and five revolutions and subsequent annealing at (**a**) 373 K, (**b**) 473 K, (**c**) 573 K for 10 min. TEM observations were performed at RT (**a**–**c**), SAED at (**a**—left SAED) RT and (**a**–**c**—right SAED) 120 K.

As was already noted, XRD analysis showed that the Ni_50_Mn_28.5_Ga_21.5_ alloy subjected to HPT at 5 GPa and five revolutions, in accordance with TEM data, had a γ-FCC structure of type *A*1 (Figure 1e,f), which was preserved during cooling in situ to 130 K. However, all the Bragg reflections were noticeably broadened. Annealing at 373 and 473 K did not change the described nanostructural γ state of the HPT-ed alloy (Figure 5a,b). After annealing at 573 K, the diffuse halo on the SAED patterns had almost disappeared, indicating the realization of a thermally activated process of devitrification of the amorphous component (Figure 5c).

**Figure 6:**
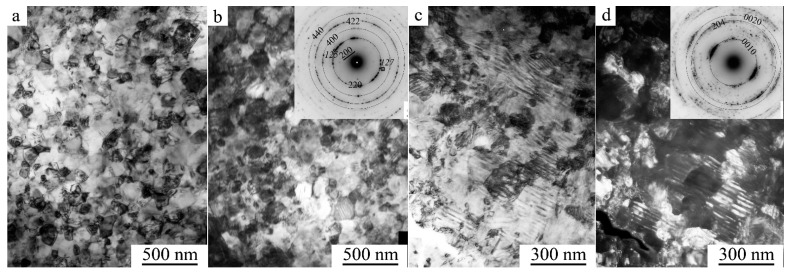
(**a**,**c**) Bright- and (**b**,**d**) dark-field TEM images of the microstructure and corresponding SAED patterns (in the inserts) of the Ni_50_Mn_28.5_Ga_21.5_ alloy subjected to HPT at 5 GPa and five revolutions and subsequent annealing at 623 K for 10 min. Observations were performed at (**a**,**b**) RT and (**c**,**d**) 120 K.

Annealing at 623 K led to more noticeable structural changes in the alloy (Figure 6). As a result of the primary recrystallization, the size of the nanograins significantly increased in the wide range of 50–150 nm. However, in the SAED pattern of the insert in Figure 6b, superstructural reflections distributed by the rings (of types 100 from the *B*2, or 111 and 200 from the *L*2_1_ phases) were not practically resolved, excluding individual marked reflections (200 *L*2_1_, 125 10*M*, 127 14*M*). Cooling to 120 K revealed the presence of 10*M* martensite reflections (for example, see Figure 6d, insert). Figure 6c,d shows bright- and dark-field TEM images of twinning inside martensitic grains larger than 100 nm. It can be assumed that TMPT occurred in *L*2_1_ grains.

**Figure 7:**
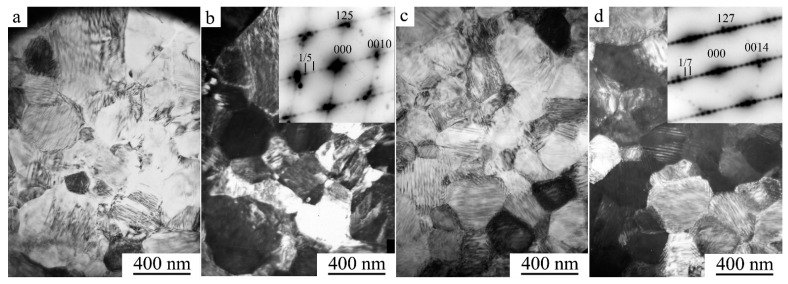
(**a**,**c**) Bright- and (**b**,**d**) dark-field TEM images of the microstructure and corresponding SAED patterns (in the inserts) of the Ni_50_Mn_28.5_Ga_21.5_ alloy subjected to HPT at 5 GPa and five revolutions and subsequent annealing at 773 K for 10 min. Observations were performed at (**a**,**b**) RT and (**c**,**d**) 120 K.

Recrystallization annealing at 773 K caused more noticeable grain growth and, obviously, a complete restoration of the perfect metastable *L*2_1_ superstructure. The grain sizes did not exceed 400 nm. As a result, 10*M* martensite in the UFG state was observed at RT (Figure 7a,b). When cooled by the in situ TEM method, the alloy experienced a second TMPT of the 10*M* → 14*M* type (Figure 7c,d). After annealing at 873 K, the grain sizes did not exceed 1 micron. The nanotwin morphology of 14*M* martensite inherited from 10*M* martensite also differed, as in the case of 10*M*, by the exceptionally single-packet character of individual grains.

Thus, in the Ni_50_Mn_28.5_Ga_21.5_ alloy, it is established that as the pressure value and the degree of shear SPD increased, a baro- (pressure-provided) and deformation-induced stepwise structural-phase transformation occured according to the scheme 10*M* → 2*M* → *B*2 → *A*1, and at the same time cascade-wise atomic disordering occured. The effect of stabilization of the nanostructured phases *B*2-BCC, *A*1-FCC, and *L*2_1_ austenite with respect to TMPT was found at a critical grain size of up to 100 nm when the alloy was cooled to 120 K. The obvious reasons for the suppression of TMPT in Ni-Mn-Ga-based Heusler alloys and the like, as well as in titanium nickelide alloys, are (i) deformation-induced reduction in the size of nanograins below the critical level, (ii) atomic disordering, (iii) amorphization, and (iv) the detected sequential SPT 10*M* → 2*M* → *B*2→*A*1. The subsequent recrystallization annealing ensures (i) the growth of grains characteristic of the UFG state, (ii) restoration of the atomic order inherent to the *L*2_1_ type, and, as a result, (iii) restoration of reversible TMPTs.

The baro-induced transition 10*M* → 2*M* occurred (i) under the influence of high pressure at 3 and 12 GPa with an increase in the value of the specific volume, as well as (ii) in the nanophases *B*2 and *A*1 formed in the course of HPT at high pressure of 3 and 5 GPa (at *n* = 2 or 5, respectively, see Table 1). Consequently, the appearance of the observed phases in the sequence 10*M* → 2*M* → *B*2→*A*1 was due not so much to the pressure as to the actual intense SPD (for example, *e* = 7 after five revolutions at the site of half of the disk radius). Both pressure-induced (under the influence of high pressure) and deformation-induced (under shear SPD) phase transitions were accompanied by atomic and structural disordering. It is this phenomenon that actually determined the key physical nature of the detected phase transitions. It is obvious that in this case, annealing, which restored the atomic order and type of superstructure, as well as grain size above the critical level with a significant decrease in the number and density of defects in their internal structure, led to the implementation of reversible TMPTs inherent in the alloy, as found in the investigations conducted.

**Figure 8:**
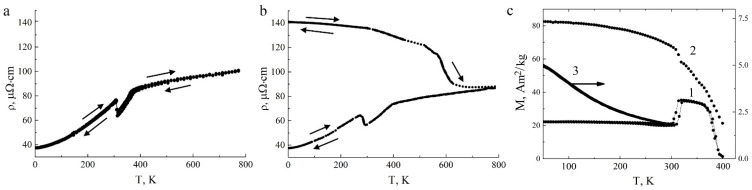
(**a**,**b**) Temperature dependences of electrical resistivity *ρ*(*T*) and (**c**) magnetization *M*(*T*) of Ni_50_Mn_28.5_Ga_21.5_ alloy in the initial cast state or (**c**) after HPT at five revolutions and 5 GPa (*curves* 1 at 0.8 MA/m, 2 and 3 at 4 MA/m). *Curve* 3, right ordinate axis.

A more complete physical interpretation of data obtained for the first time on new deformation-induced phases and structural-phase transformations in the Ni_50_Mn_28.5_Ga_21.5_ alloy was provided by using the results of temperature measurements of electrical resistivity *ρ*(*T*) in the initial cast state and after HPT (Figure 8a,b). In the figure, the arrows show the directions of the temperature change cycles during measurements of *ρ*(*T*), starting from RT to 4.2 K during cooling, then up to 800 K during heating, reverse cooling to 4.2 K and again reheating to RT. First, it can be seen that the dependence *ρ*(*T*) changed after the HPT. Namely, the alloy subjected to HPT had high values of *ρ* = 140 µΩ·cm below 400 K, and its low-temperature behavior was characterized by an anomalous negative temperature coefficient of resistivity (TCR) compared to the normal TCR for the original cast prototype alloy. The value of the residual electrical resistivity *ρ*_0_ at a temperature of 4.2 K in the studied HPT samples showed an almost four-fold increase compared to the value *ρ*_0_ of the cast alloy. 

Second, in the high-resistivity HPT alloy, the characteristic anomalies inherent to the TMPTs were not observed on the curves *ρ*(*T*). Finally, subsequent heating in the interval from 600 to 800 K led to a decrease in *ρ*(*T*) almost to the value of the initial cast alloy, and then the appearance of a narrow hysteresis loop due to the reversible TMPT (Figure 8b). The measured critical temperatures of TMPTs are given in Table 2. The behavior of *ρ*(*T*) during heating after cooling, shown in Figure 8b, clearly reveals two stages: relaxation of elastic-plastic distortions (recovery) in the temperature range from RT to 550 K and recrystallization in the range 550–800 K.

Figure 8c shows the results of measurements of the magnetic properties of the Ni_50_Mn_28.5_Ga_21.5_ alloy in the cast state and after HPT. When analyzing the magnetization of *M*(*T*), measurements were carried out starting from RT down to 4.2 K when cooling, then when heating to 400 K (*curves* 1 and 2) or up to RT (*curve* 3). From the data obtained, it can be seen that the application of a strong magnetic field *H* = 4 MA/m increased the temperatures of *M*_s_ and *M*_f_ by 10 and 5 K, respectively (Table 2). Also, attention is drawn to the sharp decrease in the magnetization of *M(T)* at RT in the HPT alloy compared to *M(T)* of the prototype alloy. However, when cooled to 4.2 K, the value of *M(T)* increased noticeably (Figure 8c, *curve* 3). The magnetization *M(T)* on cooling approached the value 60 A·m^2^/kg, whereas for the alloy in the initial state the value of its magnetization *M(T)* was greater than 80 A·m^2^/kg. Thus, analysis of the results of the structural studies and physical measurements shows that when the temperature drops below RT, despite the lower magnetization value, the HPT-ed γ alloy is in a partially magnetically ordered state, obviously genetically related to the original *L*2_1_ alloy structure.

In conclusion, we note that the HPT-ed alloy, like its prototype alloy, experienced brittle fracture–destruction when bending. At the same time, after the formation of the UFG structure during annealing at 773 K and 873 K, the alloy became sufficiently plastic to bend and under subsequent heating experienced reversible deformation into a flat shape, showing SM effect.

## 4. Summary and Conclusions

In this work, we established the effect of SPD on Ni_50_Mn_28.5_Ga_21.5_ alloy using high-pressure uniaxial compression (HPC) and torsion (HPT). The following main conclusions are made:

1. It was found that with an increase in pressure from 3 to 12 GPa, a metastable long-period 10*M* martensitic structure underwent a baro-induced transformation into a non-modulated 2*M* structure.
2. HPT radically refined the polycrystalline grain structure to a nanocrystalline, partially amorphized state at the grain junctions. As the pressure increased (up to 5 GPa) and the degree of deformation achieved the value of true deformation *e* = 7 (after five revolutions), a deformation-induced atomic disordering evolved and a stepwise structural-phase transformation occurred according to the scheme 10*M*→2*M → B*2→*A*1.
3. HPT shear of five revolutions under a pressure of 3 GPa provided incomplete deformation-induced atomic disordering and a stepwise structural-phase transformation according to the scheme 10*M* → 2*M* → *B*2 + *A*2.
4. Annealing at a temperature of 573 K caused the amorphous phase to decompose, and recrystallization annealing at 773–873 K entailed the formation of the *L*2_1_ UFG structure characterized by a narrow-hysteresis TMPT with critical temperatures close to those characteristic of the cast prototype alloy.
5. It was found that the dimensional effect of complete stabilization of a nanostructured alloy with respect to TMPT when cooled to 120 K was realized at a grain size of less than 100 nm.
6. The electrical resistivity of the HPT-ed γ alloy increased significantly during cooling, demonstrating a negative value of temperature coefficient of resistivity. In this case, the alloy was in a partially magnetically ordered high-resistivity state, the magnetization of which increased significantly in strong magnetic fields at low temperatures.
7. It can be assumed that the creation of a UFG structure in the alloy allowed its ductile properties to increase to those necessary for the implementation of an SM effect.

## Figures and Tables

**Table 1:** Type, crystal structure parameters (*a*, *b*, *c*), and specific atomic volume (*V*) of phases in the alloy Ni_50_Mn_28.5_Ga_21.5._

Type of Structure	Space Group	*a*, nm	*b*, nm	*c*, nm	*V*, nm^3^
10*M*/orthoromb.	Pnnm	0.422	0.558	2.098	0.01235072
2*M/*tetragon.	I4/mmm	0.5512		0.6562	0.01246048
*B*2/BCC	Pm3m	0.2923			0.01248695
γ-*A*1/FCC	Fm3m	0.3684			0.01249968

**Table 2:** Critical temperatures of the TMP-transformed alloy Ni_50_Mn_28.5_Ga_21.5_.

Condition	*M_s_*, K	*M_f_*, K	*A_s_*, K	*A_f_*, K	Δ*M*	Δ*A*	Δ*T*	*T*_C_, K
Cast alloy/*ρ*(*T*), Figure 8a	311	304	311	315	7	4	6	395
Cast alloy/*M*(*T*), Figure 8c, *curve* 1	311	302	312	322	9	10	10	395
Cast alloy/*M*(*T*), Figure 8c, *curve* 2	319	309			10			395
HPT at 5 GPa at 5 revolutions/*ρ*(*T*), Figure 8b	287	276	282	293	11	11	6	400
